# Cupric Oxide (CuO) Oxidation Detects Pyrogenic Carbon in Burnt Organic Matter and Soils

**DOI:** 10.1371/journal.pone.0151957

**Published:** 2016-03-24

**Authors:** Jeff Hatten, Miguel Goñi

**Affiliations:** 1 Department of Forest Engineering, Resources & Management, Oregon State University, Corvallis, Oregon, United States of America; 2 College of Earth, Ocean, and Atmospheric Sciences, Oregon State University, Corvallis, Oregon, United States of America; Old Dominion Univ., UNITED STATES

## Abstract

Wildfire greatly impacts the composition and quantity of organic carbon stocks within watersheds. Most methods used to measure the contributions of fire altered organic carbon–i.e. pyrogenic organic carbon (Py-OC) in natural samples are designed to quantify specific fractions such as black carbon or polyaromatic hydrocarbons. In contrast, the CuO oxidation procedure yields a variety of products derived from a variety of precursors, including both unaltered and thermally altered sources. Here, we test whether or not the benzene carboxylic acid and hydroxy benzoic acid (BCA) products obtained by CuO oxidation provide a robust indicator of Py-OC and compare them to non-Py-OC biomarkers of lignin. O and A horizons from microcosms were burned in the laboratory at varying levels of fire severity and subsequently incubated for 6 months. All soils were analyzed for total OC and N and were analyzed by CuO oxidation. All BCAs appeared to be preserved or created to some degree during burning while lignin phenols appeared to be altered or destroyed to varying extents dependent on fire severity. We found two specific CuO oxidation products, o-hydroxybenzoic acid (oBd) and 1,2,4-benzenetricarboxylic acid (BTC2) that responded strongly to burn severity and withstood degradation during post-burning microbial incubations. Interestingly, we found that benzene di- and tricarboxylic acids (BDC and BTC, respectively) were much more reactive than vanillyl phenols during the incubation as a possible result of physical protection of vanillyl phenols in the interior of char particles or CuO oxidation derived BCAs originating from biologically available classes of Py-OC. We found that the ability of these compounds to predict relative Py-OC content in burned samples improved when normalized by their respective BCA class (i.e. benzene monocarboxylic acids (BA) and BTC, respectively) and when BTC was normalized to total lignin yields (BTC:Lig). The major trends in BCAs imparted by burning persisted through a 6 month incubation suggesting that fire severity had first order control on BCA and lignin composition. Using original and published BCA data from soils, sediments, char, and interfering compounds we found that BTC:Lig and BTC2:BTC were able to distinguish Py-OC from compounds such as humic materials, tannins, etc. The BCAs released by the CuO oxidation procedure increase the functionality of this method in order to examine the relative contribution of Py-OC in geochemical samples.

## Introduction

Fire can significantly reduce the amount of carbon at the ecosystem level, and leave residual organic materials, such as black carbon and poly-cyclic aromatic hydro-carbons (PAHs; Table 1). Black carbon is a heterogeneous, aromatic, and C-rich residue [1] and PAHs are compounds composed of several fused benzenoid rings [2]. Together, the broad class of compounds produced as a result of incomplete combustion, including black carbon and PAHs, is referred to as pyrogenic organic carbon (Py-OC) in this paper. Pyrogenic organic carbon has been found to make up a large proportion of organic matter in soils and sediments from a variety of environments [3–6]. Because fire regimes (fire frequency and severity) have changed and will continue to change as a result of climate change and fire suppression [7, 8], it is critical to understand the role of wildfire in altering organic matter composition and carbon stocks in order to predict changes in globally-relevant Py-OC dynamics.

**Table 1:** List of abbreviations used in this manuscript (alphabetical order).

Abbreviation	Name
%IY	percent initial yield
%RY	percent relative yield
3,5-Bd	3,5-dihydroxybenzoic acid
BA	benzene monocarboxylic acids
BCA	hydroxy benzoic acid
Bd	benzoic acid
BDC	benzene dicarboxylic acids
BPCA	benzene poly-carboxylic acids
BTC	benzene ditricarboxylic acids
BTC1	1,2,3-Benzenetricarboxylic acid
BTC2	1,2,4-benzenetricarboxylic acid
BTC3	1,3,5-Benzenetricarboxylic acid
COP	CuO oxidation product
CP	cinnamyl phenols
Lig	Lignin
mBd	m-hydroxybenzoic acid
mBDC	m-Benzenedicarboxylic
N	nitrogen
NMR	nuclear magnetic resonance
oBd	o-hydroxybenzoic acid
oBDC	o-Benzenedicarboxylic acid
OC	organic carbon
PAH	poly-cyclic aromatic hydro-carbon
pBDC	p-Benzenedicarboxylic
Py-OC	pyrogenic organic carbon
SP	syringyl phenols
Vd	vanillic acid
Vl	vanillin
VP	vanillyl phenols

Assessing the contribution of Py-OC to organic carbon (OC) pools has been conducted through various methods that examine *specific* compound classes (e.g. black carbon-only, PAHs-only). The CuO oxidation procedure yields products from *multiple* biochemical precursors, including lignin, cutin, fatty acids, and specific amino acids [9–11], allowing inferences to be made on the source of organic matter in soils and sediments. CuO oxidation also yields a suite of benzoic acid products that have been utilized to measure the relative degradation state of organic matter [12–14]. In addition, upon CuO oxidation, condensed aromatic structures such as PAHs and/or black carbon yield a series of benzene products with one or more carboxylic acid groups that may be used as tracers for Py-OC[15]. In their study, Dickens et al. [15] showed that thermal alteration of pine and alder wood yields highly elevated yields of benzene carboxylic acid products (BCAs). However, unburned samples (such as tannic acid, vascular plant tissues, and algae) also yielded some of the BCA compounds, leading the authors to question whether CuO oxidation products could be used to quantify Py-OC in natural and artificial samples. In this paper, we revisit this question and show that the yields and compositions of BCA products, especially when compared to the yields and compositions of other biomarkers such as lignin-derived phenols, provide information about the contribution of Py-OC and in determining the alteration of organic matter in soils. Specifically, we examined Py-OC in soils from microcosms burned in under controlled conditions at varying levels of fire severity and subjected to laboratory incubations [16, 17]. In combination with published studies[15], we use these data to evaluate the production and consumption of Py-OC during burning and post-fire degradation.

## Materials and Methods

### Burning and Incubation Experiments

**Fig 1:**
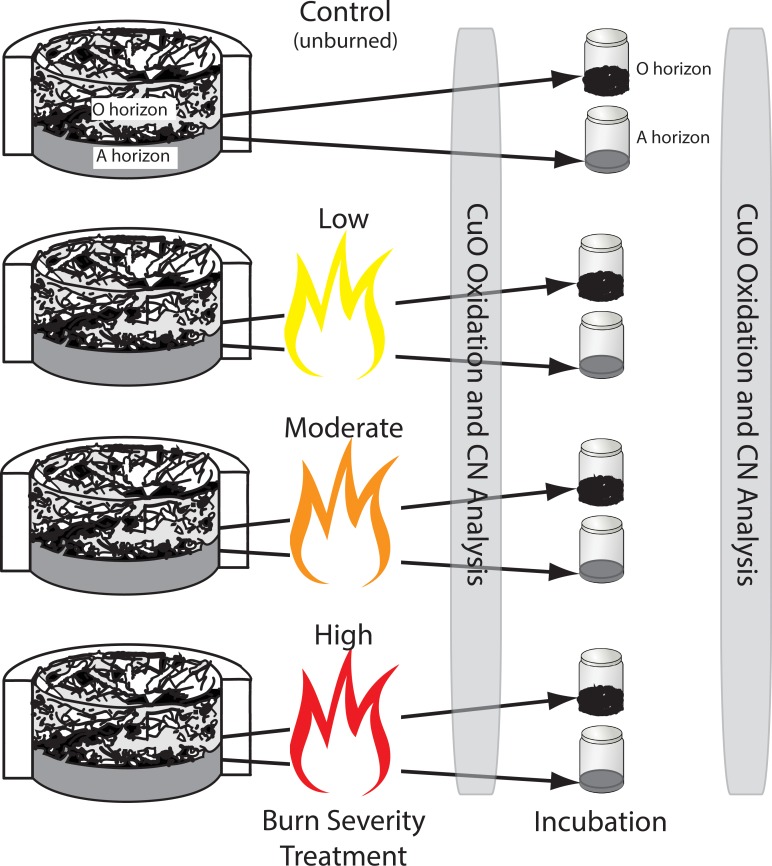
Schematic of burn severity treatments and incubation.

Details of the laboratory burns can be found in Hatten and Zabowski [16] and Hatten and Zabowski [17], while details regarding the soils that were used in this experiment can be found in Hatten et al. [18] and Zabowski et al. [19] and Fig 1. Briefly, O and A horizons were collected from a ponderosa pine forest in Eastern Washington (47°27’00”N/ 120°37’54”W; 1070 m elevation). These soils were collected from a fire prone environment and likely contain some Py-OC created by past fires, including the last major fires that affected the region in 1929 and 1890 [18]. Char was evident in the A horizon; however, none was apparent through visual examination in the O horizon. In a previous study we found that the unburned O-horizon did contain some Py-OC as measured by the chemo-thermal oxidation method, which is only able to isolate and quantify the most recalcitrant soot fraction of the Py-OC continuum [20, 21]. The Py-OC in the unburned O-horizon has probably been introduced into the O-horizon through bioturbation at the O/A interface and atmospheric deposition of Py-OC from nearby fires. After air drying, O horizons were separated into Oi (unaltered pine litter) and Oe (slightly altered organic material or duff). Mineral soil (A horizon) was sieved to 2 mm, homogenized, and stored in airtight containers.

A cylindrical form (36 cm diameter) was used to reassemble one soil microcosm for each fire severity treatment and associated control. Sieved A horizon material was added to a depth of 2 cm and tamped to a bulk density of 0.8 g cm^-3^. Air-dried Oe horizon was placed on top of the mineral material to a depth of 4 cm at a bulk density of 0.09 g cm^-3^ and air-dried Oi horizon was placed on top of the Oe material to a depth of approximately 2 cm at a bulk density of 0.03 g cm^-3^.

Flaming combustion was induced with the short (ca. 15 sec) application of a propane torch to the surface of the O horizon. Fire intensity was controlled by adjusting fuel moisture and applying heated air. An O horizon with 18% moisture content was used for the low-severity treatments. To achieve moderate- and high-severity burn levels the fuels needed to be dried (using a convection oven at 40°C) to 9% fuel moisture content. For the high-severity burns a heat gun was positioned 30 cm above the surface of the O horizon and aimed at the center of the circular form. Heated air was supplied until completion of flaming combustion.

The temperature of the burning soil microcosms was monitored with three thermocouples connected to a datalogger that recorded temperature once every minute. The thermocouples were placed on the surface of the O horizon, at the interface of the O horizon and mineral soil (0 cm), and 2 cm into the mineral soil. After burning, the soil columns were disassembled into O and A (0–1 cm) horizons. The A horizon from 1–2 cm depth was not analyzed for this study. To control for additions of organic matter from the O horizon into the A horizon, control columns were also assembled, but not burned, and then disassembled similarly to the burn treatments.

Mass loss was determined by recording the mass of O horizon before and after burning. Since the mass loss from the A horizon could not be directly measured we calculated it from organic matter content. We assumed that all mass loss that occurred from the A horizon during burning was a result of combusted organic matter. Loss on ignition (550°C for 6 hours) was used to measure organic matter in both horizons.

Following laboratory burns, the O and A horizons were incubated at 24°C for 180 days. The rate of CO_2_ production (i.e. C-mineralization rates) of each incubated soil were analyzed and described in Hatten and Zabowski [22]. Briefly, 100 g of air-dried mineral soil or 10 g of organic soil was placed into a 2-L canning jar. Inoculating solution was produced by shaking 400 g of A horizon soil with 4 L of deionized water for 24 h and the solution separated from the residual soil using qualitative filter paper with a vacuum applied to a Buchner funnel. The A horizons were moistened with enough inoculating solution to bring the moisture content to 35% gravimetric moisture content (ca. 85% of field capacity), while the O horizons were moistened with solution to achieve 100% gravimetric moisture content. Approximately 35 and 10 mL of inoculant were added to organic and mineral soils, respectively. The 2-L canning jar was capped with an airtight lid and gasses in the incubation vessel were renewed by removing the lid for at least 15 min and allowing a fan to circulate air over the chambers. Carbon dioxide was adsorbed in NaOH traps, which were collected at times of gas exchange, and estimated gravimetrically after drying. At the time of gas exchange, soil gravimetric moisture content was brought up to 35 and 100% for A and O horizons, respectively, using deionized water. For the first 4 weeks, gasses were exchanged and the soil moisture adjusted weekly. The frequency of gas exchange lengthened to 2 weeks. Mass losses were not measured during incubations; so we used an alternative approach [23] to estimate loss/production of individual constituents (see below).

### Elemental and Py-OC Analyses

The contents of OC and nitrogen (N) in all samples were measured via high-temperature combustion after removal of inorganic carbonates by vapor-phase acidification [24–26]. Briefly, subsamples of ground and homogenized soil were placed in 8x5mm silver capsules, placed in a desiccator, and exposed to concentrated HCl fumes for 24–36 h to remove any inorganic carbon [24]. After removing excess acid by oven drying for 24h, the OC and N contents of the soils were determined by high-temperature combustion using a Thermoquest NC-2500.

Ground and homogenized O and A horizons were analyzed by alkaline CuO oxidation [27, 28] to obtain the yields of a variety of products derived from burned and unburned organic matter. Briefly, sub-samples containing 2–5mg OC were extracted with 50mg of ammonium iron (II) sulfate hexahydrate. Alkaline CuO oxidations were carried out under an N_2_ atmosphere with oxygen-purged 2N NaOH at 150°C for 90minutes using a microwave digestion system. Ethylvanillin and trans-cinnamic acid were added as recovery standards after digestion and the solution was acidified to pH 1 with concentrated HCl. Samples were then liquid-liquid extracted with ethyl acetate. Anhydrous sodium sulfate was added to the ethyl acetate phase to remove water and the extracts were evaporated to dryness under a stream of N_2_ in a hot water bath heated to 40°C. The CuO reaction products were redissolved in pyridine and derivatized with bis- trimethylsilyl trifluoroacetamide+10% trimethyl-chlorosilane. The yields of individual lignin oxidation products and benzene carboxylic acids (BCAs) were quantified by gas chromatography–mass spectrometry. The compounds were separated chromatographically in a 30mx250 mm DB1 (0.25 mm film thickness) capillary gas chromatography column, using an initial temperature of 100°1C, a temperature ramp of 4°C min^-1^ and a final temperature of 300°C. The mass spectrometer was run in electron impact mode, monitoring positive ions from a range of 50–650amu. External calibration standards were determined for all compounds using ions specific to each chemical structure. The calibrations, which were performed on a regular basis to test the response of the gas chromatographer–mass spectrometer, were fit to either a linear or polynomial function (r^2^>0.99) over the concentration ranges measured in the samples.

In general we quantified two broad classes of reaction products (lignin and benzene carboxylic acids or BCAs) that we hypothesize as being unaltered and altered by burning, respectively. Products of unaltered, hereafter called unburned, organic matter included those derived from lignin (Lig = VP+SP+CP); where VP = vannilyl phenols (vanillin (Vl) + acetovanillone + vanillic acid (Vd)), SP = syringyl phenols (syringealdehyde + acetosyringone + syringic acid), and CP = cinnamyl phenols (p-coumaric acid + ferulic acid). The reproducibility of the analysis was determined on selected duplicate samples. We found that individual compounds within the Lig class were able to be reproduced within 0.4–1.4% (mean = 0.9%) of the soil mass normalized values.

Reaction products of altered, hereafter called burned, organic matter were represented by three classes of BCAs. We quantified the yields of benzoic and hydroxybenzoic acids with one carboxylic functional group (BA = benzoic acid (Bd) + *o*-hydroxybenzoic acid (oBd) + *m*-hydroxybenzoic acid (mBd) + 3,5-dihydroxybenzoic acid (3,5-Bd)), two carboxylic acid functional groups (BDC = *o*-Benzenedicarboxylic acid (oBDC) + *m*-Benzenedicarboxylic acid (mBDC) + *p*-Benzenedicarboxylic acid (pBDC)), and three carboxylic acid functional groups (BTC = 1,2,3-Benzenetricarboxylic acid (BTC1) + 1,2,4-Benzenetricarboxylic acid (BTC2) + 1,3,5-Benzenetricarboxylic acid (BTC3)). The reproducibility of the analysis was determined on selected duplicate samples. We found that individual compounds of the BA, BDC, and BTC classes were able to be reproduced within 0.6–6.3% (mean = 2.2%), 0.8–6.3% (mean = 2.8%), and 1.2–3.8% (mean = 2.2%), respectively.

Total CuO oxidation product yield (COP) was calculated as the sum of all quantified CuO oxidation products (VP, SP, CP, BA, BDC, and BTC). All CuO oxidation products were quantified using external calibration standards. The calibrations were performed on a regular basis and were highly linear (R^2^>0.99). The soil normalized values of individual biomarkers are listed in S1 Table.

### Data Analyses

Trends in constituents caused by burning and incubation were assessed using Pearson’s correlation (R) before and after incubation. We used the maximum temperature achieved during the burning treatment as the indicator for fire severity. We also used this approach when assessing data from Dickens et al. [15]. Care must be taken when exploring trends in data sets with small samples sizes. We considered a trend significant when the p-value was less than 0.05. This level of significance corresponded to an R>0.950 when n = 4, or highly linearly correlated and a probability of random occurrence (i.e. type I error) of 5%. Furthermore, the relationship between burn temperature and constituent may have been non-linear; therefore, by assuming a linear relationship, we are taking a conservative approach when determining the trend between burn temperature and a constituent.

Mass-normalized compound yields underrepresent losses and over-represent the production of constituents since they do not take into account mass loss occurring during the experimental treatment [29]. Thus, in our study, mass-normalized yields alone do not provide an accurate representation of the quantitative loss, preservation or creation of individual compounds due to the burning and incubation processes. To address this issue, for each constituent we calculated the proportion of initial material remaining in the sample using the formula %IY = [(Yt/Yc) * (1 = (%ML/100))] * 100. Where %IY is the percent of initial yield after treatment, Y_t_ and Y_c_ are the constituent yield of treated and control or pre-incubation soils, respectively, and %ML represents the percent mass loss incurred in the sample. Mass loss was measured for burned O horizons and assumed to be negligible for burned A horizons. According to this formulation of %IY, constituents with < 100% were lost from the sample (due to combustion, mineralization, or alteration) during burning or incubation, whereas those with > 100% were produced. Components with a %IY of 100% behaved conservatively, reflecting no changes in overall yields during burning.

Mass loss was not recorded during the incubation and therefore %IY cannot be used to assess the relative response of the constituents to the incubation. We used COP to normalize for differences in the total yield of compounds. While it is thought that some components of Py-OC are more unreactive than lignin phenols [30], some of the products we are examining (i.e. BA and BDC) have been hypothesized to be products of organic matter degradation [12, 14]. After incubation the relative yield (%RY) of each constituent from each sample was calculated using the formula %RY = [(Y_i_/Y_b_) / (COP_i_/COP_b_)] * 100. Where %RY is the percent of relative yield after the incubation, Y_b_ and Y_i_ are the constituent yields of post-burn and incubated or pre-incubation soils, respectively, and COP_b_ and COP_i_ are the total CuO oxidation product (lignin and BCA COPs) yields of post-burn and incubated or pre-incubation soils. According to this calculation constituents with %RY>100% are either produced or display lower reactivity than the average COP during incubations, whereas those with %RY < 100% are more reactive and consumed preferentially during incubations.

To determine if a constituent was more or less resistant to burning or incubation than bulk OC we utilized a paired sample t-test between the constituent’s %IY and %RY and those calculated for bulk OC in both both O and A horizons.

## Results

### Compositional Changes as a Result of Fire Severity

The three burn experiments revealed significant alterations to OC content and composition that were correlated with increasing maximum temperature at the surface of the O and A horizons (Table 2). The low-severity burn treatments charred the Oi horizon (i.e. unaltered litter), leaving the underlying Oe horizon (i.e. duff or partially decomposed litter) visually unaltered. As expected, the low severity fire did not heat the mineral soil as drastically as the moderate and high severity burn treatments. The moderate and high severity burn treatments charred nearly the entire O horizon, while the high severity burns consumed most of the O horizon. These high rates of consumption led to higher maximum temperatures at the surface and in the mineral soil for the moderate and high severity fires. Mass loss of the O horizon (31.7–80.2%) and maximum temperature of the O (100–258°C) and A horizons (100–234°C) were significantly correlated (r = 0.987 and 0.945 with p = 0.001 and 0.015, respectively). This led to decreases of OC contents in both O and A horizons.

**Table 2:** Burn characteristics and soil normalized CuO oxidation product classes of O and A horizons from burned soil microcosms. Pearson correlation and p-value calculated on relationship between constituent and maximum temperature of horizon during burn treatment. p-values less than 0.05 are in bold.

	O Horizon						A Horizon					
	Control	Low	Moderate	High	r	*p*	Control	Low	Moderate	High	r	*p*
Mass Loss (%)	0.0	31.7	67.2	80.2	*0*.*989*	***0*.*011***	0.0	0.0	3.9	4.9	*0*.*935*	*0*.*065*
Max Temperature (°C)	15	100	252	258			15	100	213	234		
	mg g^-1^ soil											
OC	462.6	450.8	429.9	197.1	-0.654	*0*.*346*	47.4	54.9	44.6	40.5	-0.619	*0*.*381*
N	6.2	8.3	11.7	6.3	0.455	*0*.*545*	2.3	2.5	2.5	2.4	0.536	*0*.*464*
OC:N_molar_	86.5	63.4	42.8	36.4	-0.986	***0*.*014***	24.1	25.3	21.1	19.4	-0.850	*0*.*150*
V	24.14	18.59	5.88	0.79	-0.982	***0*.*018***	1.03	1.40	0.85	0.53	-0.671	*0*.*329*
S	0.48	0.55	0.14	0.04	-0.907	*0*.*093*	0.12	0.14	0.09	0.09	-0.770	*0*.*230*
C	1.75	1.28	0.47	0.12	-0.985	***0*.*015***	0.18	0.16	0.10	0.09	-0.987	***0*.*013***
Lig	26.37	20.42	6.49	0.95	-0.982	***0*.*018***	1.33	1.70	1.05	0.70	-0.724	*0*.*276*
BA	1.60	2.13	2.20	1.15	-0.103	*0*.*897*	0.23	0.29	0.27	0.26	0.427	*0*.*573*
BDC	0.64	1.00	1.38	1.07	0.868	*0*.*132*	0.08	0.06	0.10	0.12	0.757	*0*.*243*
BTC	0.42	0.68	0.96	0.53	0.577	*0*.*423*	0.05	0.07	0.11	0.08	0.827	*0*.*173*
Vd:Vl	0.46	0.55	0.66	0.87	0.883	*0*.*117*	0.54	0.89	0.97	0.62	0.385	*0*.*615*
3,5-Bd:V	0.04	0.06	0.14	0.33	0.802	*0*.*198*		0.12	0.09	0.13	0.21	0.659	*0*.*341*

While there was not a significant trend in the N concentration of the post-burn residue, there was a significant negative trend in the OC:N of the remaining organic matter in both the O and A horizons with increasing fire severity (Table 2). The slight negative trend in OC concentration led to this relationship. Carbon has been found to be less thermally stable than N which leads to an enrichment of N in post-fire residues [31–36].

In the O horizon the yields of all lignin phenols had a significant negative relationship with burn temperature, consistent with many studies that found a decrease in lignin with increasing burn temperature [37–39]. Only the cinnamyl phenols (CP) had a statistically significant relationship with maximum temperature of the A horizon. This suggests that CP may be slightly less resistant to burning than the other lignin phenols, a trend that has been observed by others [39].

The yields of both BDC and BTC were positively correlated with fire severity in O and A horizons, although the trend was not statistically significant. On the other hand, yields of BA from the O horizon decreased with burn severity, due to a high degree of consumption that may have occurred by burning at high temperatures. Indeed, the O horizons treated to high severity burning all showed lower soil-normalized BA, BDC, and BTC. Yields of all BCAs from the A horizon were positively related with burn severity, but only BTC had a statistically significant relationship.

The ratios of vanillic acid to vanillin (Vd:Vl) and 3,5-dihydroxybenzoic acid to vanillyl phenols (3,5-Bd:VP) are commonly used indicators of microbial alteration of lignin moieties [12, 14, 15] and have been found to also respond to leaching/sorption [40], microbial decay [29], and burning plant components [39, 41]. We did not find a significant relationship with burn severity suggesting that the complex mixtures of organic matter sources in our samples (i.e. field collected soil horizons) may have contributed to obscure possible effects of burning on these indicators.

**Fig 2:**
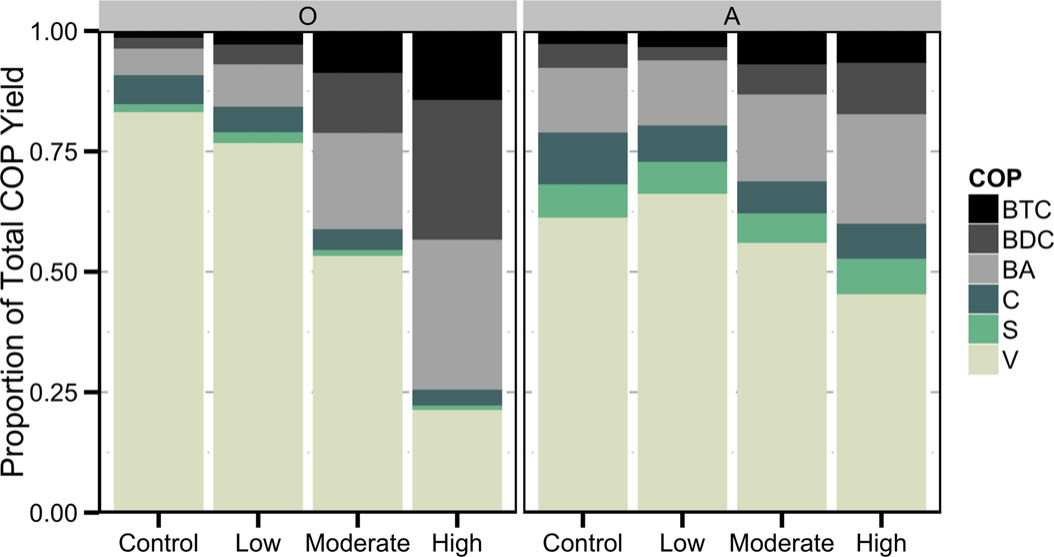
Proportion of total CuO oxidation product (COP) yield from the burn-only treatments.

Lower mass-normalized yields of lignin phenols in the burned relative to the control were contrasted by elevated BCAs yields in burned O and A horizons (Fig 2). The trend was driven by a consistent decrease in lignin phenols with increased burning severity. In fact, lignin phenols represented the majority (>56%) of all CuO oxidation products in all samples except the high severity treated O horizon (24%), The relative proportion of BCAs increases from 9% to 74% in the O horizon and 21% to 40% in the A horizon as a result of burning at the highest severity. These results suggest that lignin is consumed while BCAs are preferentially preserved or produced by burning.

**Fig 3:**
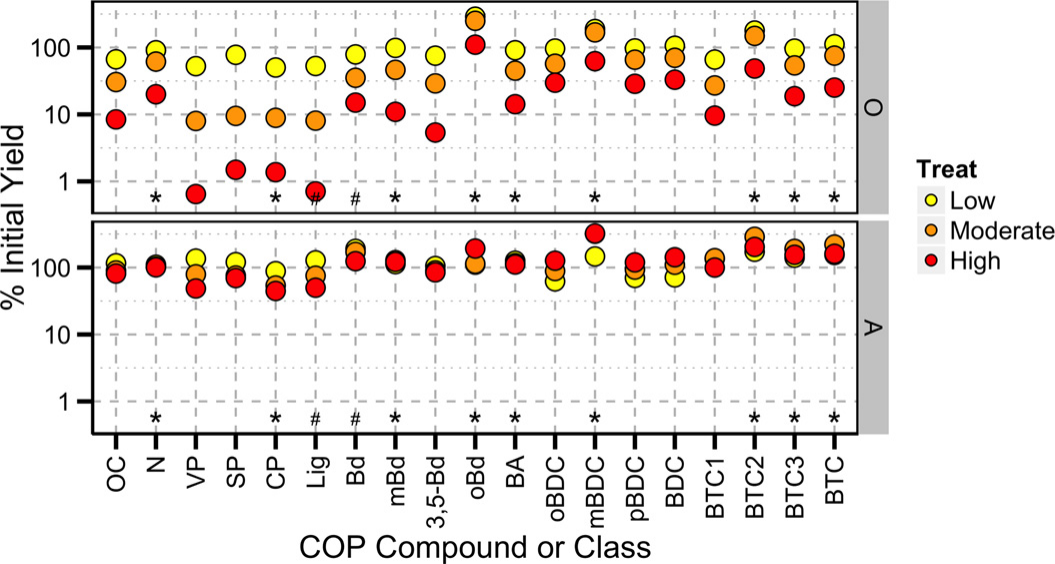
**Percent initial yield normalized for mass losses occurring as a result of burning of a) O horizons and b) A horizons from burn experiment.** Values less than 100% (horizontal dashed line) represent preferential losses while values greater than 1 represent production of those compounds as a result of burning. Asterisks and pound symbols indicate significant difference between across both horizons (N = 8) of the %IY of that constituent and bulk OC at the 0.10 (#) and 0.05 (*) **α**-value.

To correct for mass losses and compare the compositions of burned samples to those of unburned controls, we calculated the %IY of all measured organic constituents, including OC, N, lignin and non-lignin CuO products including BCAs (Fig 3). The %IY of OC and the major classes of lignin phenols and BCAs from the O horizon were near or less than 100% suggesting that these compounds were consumed by burning (Fig 3A). While the %IY of many of the lignin biomarker classes significantly decreased with increasing fire severity (Pearson’s correlation for VP, SP, CP, and Lig was R<-0.95 and p<0.05), none of the BCA classes showed a significant linear relationships with burn temperature. Many of the BCAs showed non-linear behavior that was difficult to assess with such a small sample size and also would appear as a non-statistically significant relationship in our analysis. Organic carbon, N, and individual BCA’s displayed higher %IY at all three fire-severity levels than the lignin phenols, consistent with a higher resistance to fire. Three individual compounds released from the O horizon were exceptions to the general decrease in %IY with burn severity. The %IY of oBd was 110 to 288% for all three levels of burn severity, indicating that this constituent was formed during the burning process (mass normalized data for individual compounds shown in S1 Table). Similarly, mBDC and BTC2 also appear to have been formed during the low and moderate severity burn treatments of the O horizon (%IY 149–188%). While the high severity burning of the O horizon (highest temperature) may have resulted in an overall decrease of these constituents perhaps as a result of further alteration of these Py-OC products to moieties not detected by our procedure (e.g., more highly condensed-ring structures).

The response of constituents recovered from the A horizon to burning revealed that %IY values for OC and lignin phenols classes decreased as a function of burn severity, with the lowest values (%IY < 70%) consistently observed in the high-severity treatments. Notably, the %IY values of the low-severity treatment were slightly greater than 100%, which may have resulted from the incorporation of a small amount of organic matter from the unburned O horizon [16]. It is also possible that low temperature alteration promoted enhanced yields of lignin monomers from the CuO oxidation of their macromolecuar precursor. Based on these results, it appears that bulk OC and most lignin phenols behaved conservatively under low severity conditions but were lost (especially lignin compounds) during medium- and high-severity treatments. In contrast, most BCA products displayed %IY values > 100% after medium- and high-severity burns, consistent with their net production during the pyrogenic process. Note that in contrast to OC, the %IY values for N remain ~100% for all three severity treatments indicating bulk N in the mineral soil horizons behaves conservatively through the burning process.

In both the O and A horizon, several BCAs (Bd, mBd, oBd, mBDC, BTC2, and BTC3) and classes (BA and BTC) had significantly higher %IY relative to OC. This suggests that the precursors of these compounds were preserved or created relative to bulk OC. These results are in contrast to the significantly lower %IY for the lignin phenol classes CP and Lig recovered from both horizons. Interestingly, the %IY values for N were significantly higher to bulk OC, possibly as a result of N having higher volatilization temperatures [32]. Benzoic acid (Bd) also had a significantly strong response to burning relative to bulk OC. Since Bd has many different sources in the environment [11], it may not be robust indicators of Py-OC. The %IY of mBd, oBd, mBDC, BTC2, BTC3, and the entire class of BTCs was higher than bulk OC of O and A horizons suggesting that these compounds may be robust indicators of Py-OC derived from either organic or mineral substrates.

### Compositional Changes as a Result of Burning and Decomposition

The yields of many of the CuO oxidation products (lignin and BCAs) recovered from the incubated samples were still correlated with the maximum temperature of the burn treatments (Table 3). This suggests that even after incubation, burn severity is controlling the composition of OC in both the O and A horizons.

**Table 3:** Soil normalized CuO oxidation product classes of burned and incubated O and A horizons. Pearson correlation and p-value calculated on relationship between constituent and maximum temperature of horizon during burn treatment. p-values less than 0.05 are in bold.

	O Horizon						A Horizon					
	Control	Low	Moderate	High	r	*p*	Control	Low	Moderate	High	r	*p*
	mg g^-1^ soil											
OC	438.3	465.1	415.7	258.5	-0.662	*0*.*338*	40.6	42.2	40.1	35.4	-0.655	*0*.*345*
N	9.7	11.1	11.0	6.2	-0.379	*0*.*621*	2.1	2.1	2.3	2.2	0.812	*0*.*188*
OC:N_molar_	52.7	49.0	44.2	48.7	-0.814	*0*.*186*	22.2	23.5	20.0	18.6	-0.815	*0*.*185*
V	43.33	37.79	13.74	6.13	**-0.975**	***0*.*025***	2.348	2.328	1.29	0.85	-0.934	*0*.*066*
S	1.78	1.17	0.22	0.08	**-0.999**	***0*.*001***	0.211	0.213	0.16	0.11	-0.883	*0*.*117*
C	3.59	3.05	0.84	0.45	**-0.985**	***0*.*015***	0.330	0.364	0.21	0.13	-0.872	*0*.*128*
Lig	48.69	42.01	14.80	6.66	**-0.977**	***0*.*023***	2.889	2.905	1.66	1.08	-0.925	*0*.*075*
BA	3.62	3.99	4.42	2.19	-0.259	*0*.*741*	0.457	0.478	0.47	0.42	-0.474	*0*.*526*
BDC	0.47	0.56	1.22	0.91	0.907	*0*.*093*	0.050	0.072	0.08	0.09	0.974	***0*.*026***
BTC	0.81	0.85	1.47	0.87	0.596	*0*.*404*	0.082	0.089	0.12	0.12	0.986	***0*.*014***
Vd:Vl	0.50	0.60	0.70	0.82	0.941	*0*.*059*	0.56	0.66	0.77	0.75	0.981	***0*.*019***
3,5-Bd:V	0.05	0.06	0.14	0.15	**0.975**	***0*.*025***	0.11	0.12	0.19	0.24	0.932	*0*.*068*

All of the CuO oxidation products recovered from the O horizon after burning that exhibited significant relationships between yield and maximum burn temperature continued to have significant relationships after incubation. Indeed, VP, SP, CP, and Lig had negative relationships (r<-0.975, p<0.05) with burn temperature whereas BDC had a positive relationship with burn temperature. Many of the CuO oxidation products recovered from the A horizon did not have significant relationships with burn temperature after burning or burning and incubation; however the strength of the relationship increased after incubation, probably as a result of less variability around the linear relationship between the CuO oxidation product yields and maximum burn temperature. CuO oxidation products with significant positive relationships with burn temperature included BDC and BTC (r>0.981, p<0.05).

While Vd:Vl and 3,5-Bd:V are hypothesized to increase with OC degradation [12, 14, 15] we did not observe a significant relationship with these ratios and CO_2_-C loss [16] from both O and A horizons as a result of incubation (data not shown; r>-0.730,p>0.206). It should be noted that even though they were not statistically significant all these relationships were negative suggesting that the response to burning was stronger than the response to degradation over a six-month incubation (Table 3).

**Fig 4:**
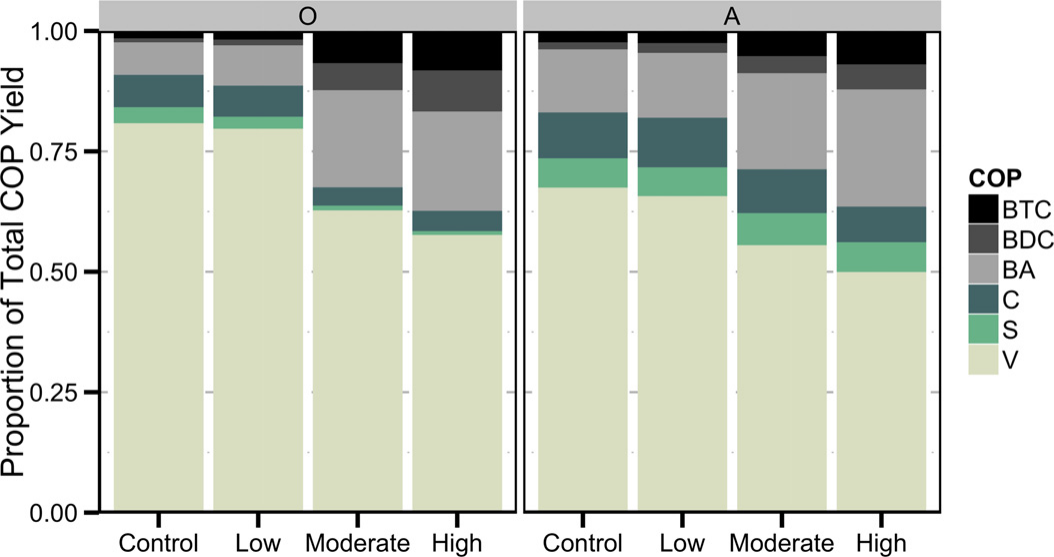
Proportion of total COP yield from post-incubation samples.

The contribution of mass-normalized lignin phenols in the burned relative to the control were contrasted by elevated BCAs in burned O and A horizons after incubation (Fig 4). While the differences were much less pronounced post-incubation the post-burn pattern is still apparent. The relative proportion of BCAs in the incubated control was 9% relative to 37% in the incubated O horizon burned at high severity and 16% and 36% in the A horizon as a result of burning at the highest severity. The post-burn pattern was still apparent after incubation and that the effects of burning were preserved through the incubation. These results suggest that these biomarkers may be robust indicators of Py-OC.

**Fig 5:**
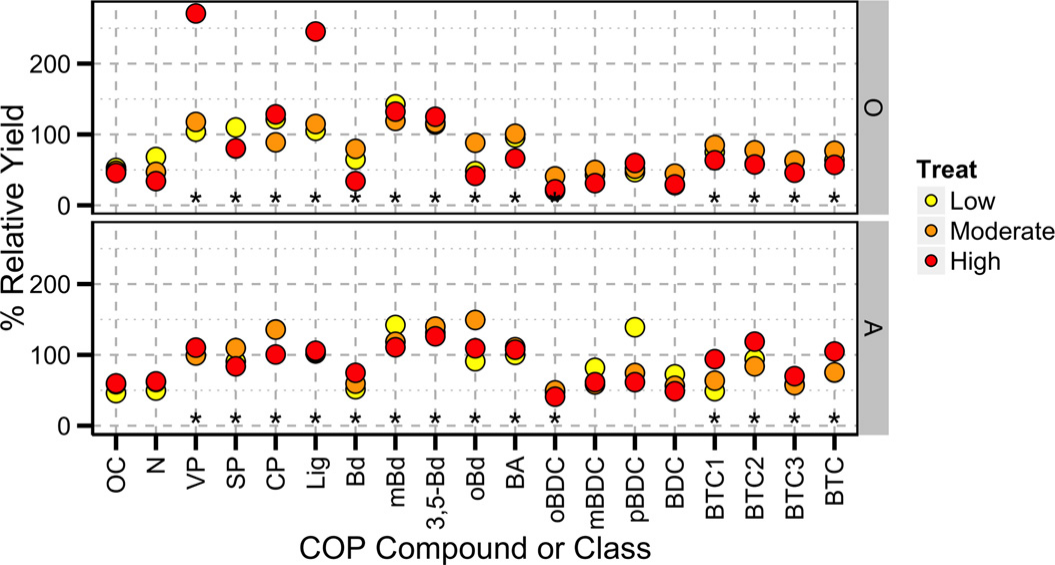
**Percent relative yield normalized by the response of the COP yield occurring as a result of incubation of a) O horizons and b) A horizons from burn experiment.** Values less than 100% (horizontal dashed line) represent losses relative to the COP yield while values greater than 100% represent production of those compounds relative to COP yield. Asterisks indicate significant difference between across both horizons (N = 8) of the %IY of that constituent and bulk OC at the 0.05 (*) **α**-value.

To assess the response of constituents to the incubation we calculated the yield of each relative to the response of total COP yield (%RY; Fig 5). These data were normalized by the pre-incubation samples for each treatment, so that we can examine how each constituent responds to the incubation. The %RY of OC decreased with incubation of O and A horizons as a result of mineralization. Aside from an outlying VP measurement of the high severity treatment the COPs of both O and A horizons behaved similarly during the incubation according to the %RY. Generally, VP, SP, and CP increased similarly cross horizons and treatments (average %RY = 114% across O and A horizons). Several BA compounds (mBd, 3,5-Bd, and oBd) and the BA class increased relative to bulk OC suggesting that these compounds may be products of organic matter decomposition and not robust indicators of Py-OC. These results are consistent with report that mBd, oBd, and 3,5-Bd are actively produced during the degradation of soil organic matter [11, 12, 14, 15]. Because of these trends, constituents such as mBd, 3,5-Bd, oBd, and the BA class will need to be used with caution when used to trace Py-OC in the environment. Several of the BCAs with 2 or 3 carboxylic acid groups (oBDC, BTC1, BTC2, BTC3) and BTC decreased relative to OC during the incubation suggesting that these compounds are not decomposition products and are likely degraded in the environment.

### OC Normalized CuO Oxidation Product Composition

Organic matter associated with soils and sediments are a mixture of organic and mineral dominated sources, therefore it is important that any measure or indicator of Py-OC be independent of the source. In addition these indicators should not be strongly altered by processes such as degradation. To examine the effects independent of matrix (i.e. organic versus mineral) and incubation and to bring in additional data from other studies we have normalized all the CuO oxidation products by the OC content (Table 4).

**Table 4:** Organic carbon normalized CuO oxidation product classes and potential Py-OC ratios. These are averages of the burned and incubated values for each burn treatment and horizon. Pearson correlation and p-values calculated across both O and A horizons (N = 8) on all samples’ relationship with maximum temperature of horizon during burn treatment. p-values less than 0.05 are in bold.

	O Horizon				A Horizon					
	Control	Low	Moderate	High	Control	Low	Moderate	High	r	*p*
	mg 100 mg^-1^ OC									
V	7.55	6.12	2.34	1.39	3.98	4.04	2.57	1.86	-0.677	**0.004**
S	0.25	0.19	0.04	0.03	0.38	0.38	0.30	0.25	-0.522	**0.038**
C	0.60	0.47	0.16	0.12	0.60	0.58	0.38	0.28	-0.678	**0.004**
Lig	8.41	6.78	2.54	1.53	4.97	5.00	3.25	2.39	-0.693	**0.003**
BA	0.59	0.67	0.79	0.72	0.80	0.83	0.89	0.91	0.171	0.526
BDC	0.12	0.17	0.31	0.45	0.15	0.14	0.21	0.28	0.773	**0.000**
BTC	0.14	0.17	0.29	0.30	0.15	0.17	0.27	0.26	0.786	**0.000**
oBd:BA	0.06	0.14	0.30	0.36	0.11	0.10	0.14	0.21	0.790	***0*.*000***
mBDC:BDC	0.17	0.23	0.27	0.20	0.17	0.19	0.26	0.23	0.458	*0*.*075*
BTC2:BTC	0.41	0.55	0.71	0.69	0.54	0.61	0.67	0.65	0.827	***0*.*000***
BTC:Lig	0.02	0.03	0.12	0.35	0.03	0.04	0.09	0.11	0.572	***0*.*021***

### Potential Py-OC Indicating Ratios

To more clearly isolate a Py-OC signal we explored ratios of singular constituents and classes of constituents (Table 4). Compound ratios are relatively insensitive to processes such as mass losses or additions and are analytically more robust than absolute yield determinations [42]. We have focused on those constituents that have the strongest response to burning as measured by %IY (oBd, mBDC, and BTC2). Individual biomarkers within each class typically behaved similarly during burning and incubation. Therefore, we normalized the individual compounds by the class (i.e. oBd with BA, mBDC with BDC, and BTC2 with BTC). We have also explored the use of a ratio that described the contribution of Py-OC relative to unburned organic matter to samples by selecting the most robust class of BCAs and normalized it by total lignin content (i.e. BTC:Lig).

Most of our selected ratios were significantly and positively related to burn temperature, mBDC:BDC was positively related to burn temperature but did not have a statistically significant linear relationship with burn temperature. oBd:BA, BTC2:BTC, and BTC:Lig had significant relationships with burn temperature across incubated and non-incubated O and A horizons, suggesting that these three ratios may be robust indicators of the charring temperature the Py-OC was created.

In general, we found significant negative Pearson correlations with maximum burn temperature and all of the OC normalized lignin classes and significant positive correlations with all of the OC normalized BCA classes, except BA. These results suggest a significant effect of burning across all sample types that is independent of matrix or incubation.

## Discussion

We examined lignin and Py-OC CuO oxidation products after burning soil microcosms (O and A horizons) at three levels of fire severity and subjecting those soils to a six-month incubation. In general, as fire severity increased lignin phenols decreased and BCAs increased. Overall, OC and the major classes of lignin phenols and BCAs were consumed by burning. However, relative to bulk OC the precursors to BCAs were either preserved or created during burning. We found that these trends were preserved after incubation with natural microbial inoculates.

### Outside Data Sources

**Fig 6:**
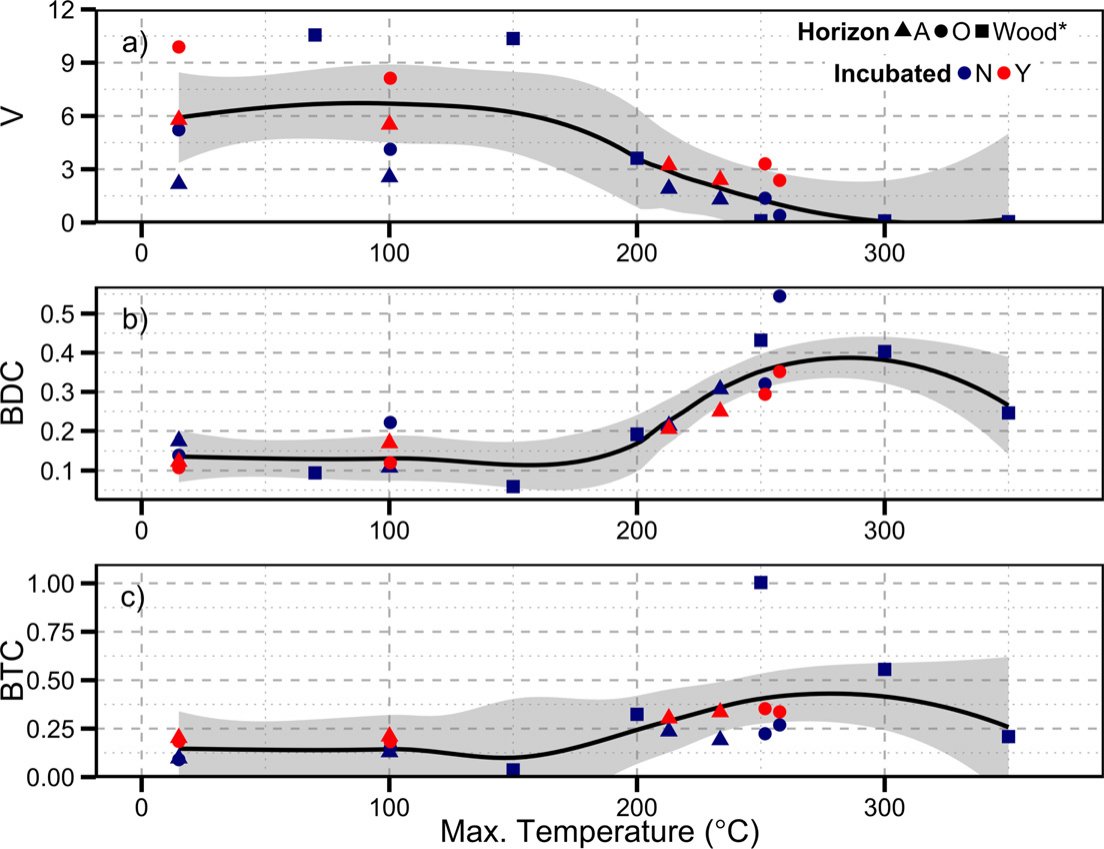
Organic carbon normalized lignin and Py-OC in relation to each horizon’s maximum temperature achieved during burning. Samples are from incubated and non-incubated O and A horizons. Lowess curve fit with 95% confidence intervals displayed in gray. *indicates pine wood samples heated in a muffle furnace and analyzed for most CuO oxidation products in Dickens et al. (2007).

**Fig 7:**
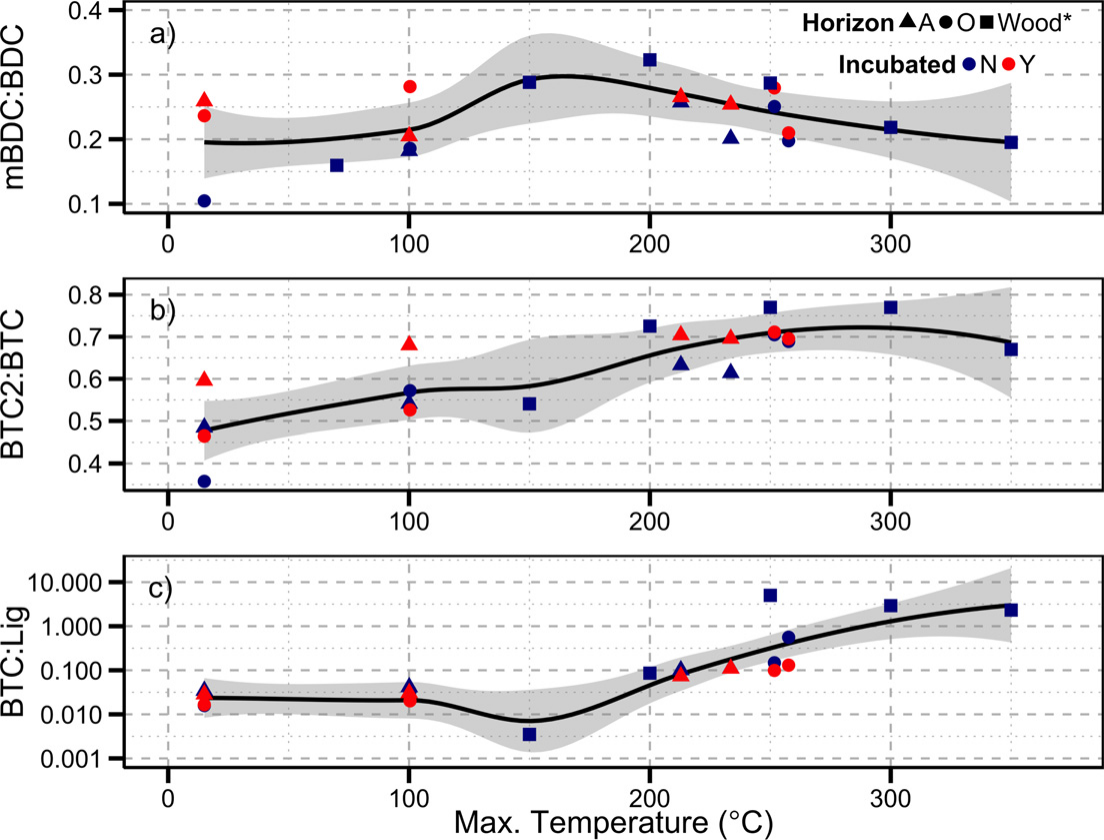
Py-OC ratios in relation to each horizon’s maximum temperature achieved during burning. Samples are from incubated and non-incubated O and A horizons. Lowess curve fit with 95% confidence intervals displayed in gray. *indicates pine wood samples heated in a muffle furnace and analyzed for most CuO oxidation products in Dickens et al. (2007).

Complete di- and tri-carboxylic benzoic acids are not often reported as products of CuO oxidation, as such we could find only one other published study that reported lignin products and BCAs in relation to burning or heating temperatures [15]. We incorporated the OC normalized lignin and BCA data reported by Dickens et al. [15] for charred pine wood into those portions of our study to demonstrate the applicability of this method across several sample types (Figs 6 & 7). Dickens et al. (2007) did not report all of the same constituents we have (e.g oBd was omitted from their report), so we have focused on those constituents that are common between the two studies. Dickens et al. (2007) report on red pine (*Pinus resinosa*) wood heated to six different temperatures and red alder (*Alnus rubra*) wood heated to 2 different temperatures. Including the wood samples from this study did not change our initial interpretation of the overall trends. We found that OC normalized VP decreased with burn temperatures above 100°C while BDC and BTC both increased. Both BDC and BTC reached a peak in yields around 280°C. It is also apparent that the source of material (O versus A horizon), and therefore any matrix effect, did not affect the OC normalized yields when the samples were burned or incubated.

Most of our selected ratios were significantly and positively related to burn temperature, mBDC:BDC was positively related to burn temperature but did not have a statistically significant linear relationship with burn temperature. oBd:BA, BTC2:BTC, and BTC:Lig had significant relationships with burn temperature across incubated and non-incubated O and A horizons, suggesting that these three ratios may be robust indicators of the charring temperature the Py-OC was created. We combined our data with burned wood from Dickens et al. (2007) and found that these ratios provide robust indicators of Py-OC across a range of sample types (Fig 7). mBDC:BDC appears to reach a peak around 190°C while BTC2:BTC appears to peak around 300°C suggesting that BTC2:BTC may be a more robust indicator of Py-OC and that these two ratios may be used in tandem to examine the relative differences burning conditions that created the Py-OC in natural samples.

### Interfering Compounds

**Fig 8:**
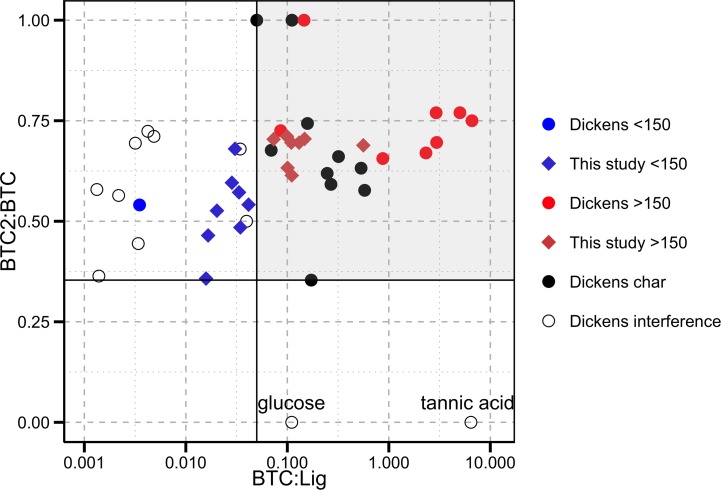
The Py-OC composition of burned and burned and incubated O and A horizons relative to all heated and burned OM that achieved a temperature >150°C and unburned and lightly heated (<150°C) OM from Dickens et al. (2007). Dickens et al. (2007) also tested whether there were unburned (non-pyrogenic) organic materials that could interfere measurement of Py-OC using the CuO oxidation method. Solid lines represents thresholds of the ratios indicative of Py-OC. Gray box bounds all samples that have been exposed to temperatures >150°C.

Benzene carboxylic acids have been shown to have sources other than Py-OC [14, 15]. Dickens et al. [15] made an extensive evaluation of whether Py-OC could be quantified using CuO oxidation-derived BCAs, and measured chars produced in the lab as well as chars that were collected from soil profiles and the boles of trees. These have been plotted with our non-incubated and incubated moderate and high severity O and A horizon samples as “>200°C” in Fig 8. They also assessed unburned pine and alder wood which has been plotted with our non-incubated and incubated control and low severity O and A horizons as “<150°C”. When these burned and unburned materials are plotted together there appears to be a distinction between the burned and unburned organic matter based on BTC:Lig (<0.04 and >0.05 for the unburned and burned material, respectively). However, Dickens et al. [15] found that glucose and tannic acid both produced lignin phenols and BTCs and therefore had ratios of BTC:Lig that would be considered Py-OC if BTC:Lig was the only indicator. Additionally, Dickens et al. [15] examined other interfering compounds including humified materials (as melanoidin), specific organic compounds (protein), organic matter (mangrove, brown and white rot, brown algae, and decomposed oak wood), and aromatic materials created as a result of geologic (graphite and bituminous coal) and fossil fuel consumption (n-hexane soot). With the use of BTC2:BTC interfering sources can be distinguished from Py-OC. Burned material and several chars of various ages can be clearly differentiated from interfering substances using a threshold of BTC2:BTC>0.35, as indicated by the values obtained from the char samples analyzed by Dickens et al. [15].

Environmental samples of soils and sediments are mixtures of materials that may have experienced burning and materials that have not. Therefore the Py-OC signature could be influenced by contributions of interfering compounds such as tannins. We can calculate the quantity of tannic acid required to raise a sample’s Py-OC signature above our threshold using a two-end member mixing model using the average BTC2:BTC and BTC:Lig composition of unburned organic matter and tannic acid. Using this approach we found that a sample would need to be composed of >70% tannic acid in order to develop a signal that would fall within the bounds of our Py-OC endmembers. Leaves can have tannin contents up to 20% [43] while wood typically has tannin contents less than 1% by weight [44]. Tannins have been shown to be roughly as degradable as the bulk organic matter, so this material is not likely to increase in relative concentration through degradation [43]. This suggests that no matter what the contribution (fresh or degraded) it is not likely that tannins can significantly influence the Py-OC signature of typical soil and sediment samples as measured by the CuO oxidation procedure.

**Fig 9:**
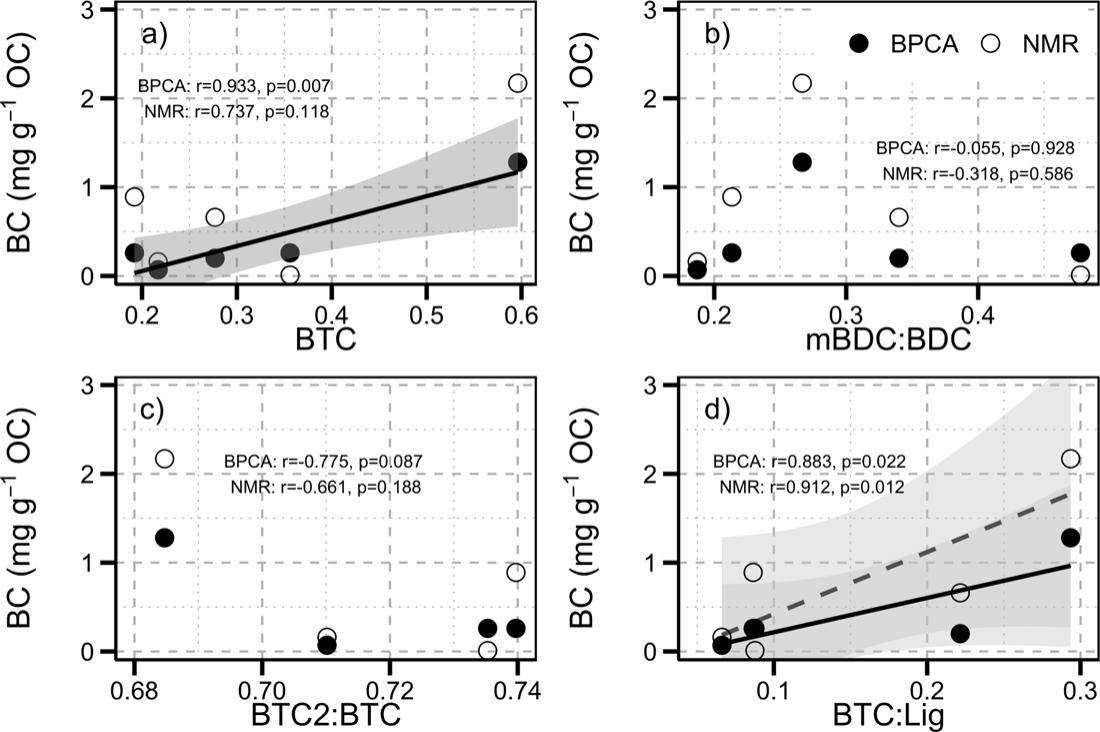
Comparison of BCAs extracted CuO oxidation to black carbon (BC) quantified using the UV-NMR and BPCA approaches. **a) BTC in mg 100g**^**-1**^ **OC, b) mBDC:BDC, c) BTC2:BTC, and d) BTC:Lig.** Significant linear relationships are shown as solid (BPCA) or dashed (NMR) lines and with 95% confidence intervals displayed in gray. The results of a Pearson correlation are displayed in text on each plot.

Our results suggest that the BTC:Lig parameter can be used to assess the overall Py-OC content while the BTC2:BTC ratio can be used to distinguish burned materials from other potentially interfering compounds. The ratio BTC:Lig may therefore correlate with some of more widely used measures of black carbon in soils. Dickens et al. [15] assessed the total CuO oxidation yield of BCAs from soils against black carbon determined by the ultra violet–nuclear magnetic resonance (UV-NMR) [45] and benzene poly-carboxylic acid (BPCA) [46] methods and found that they were not well correlated and driven by outlier samples (Fig 9). Using the data reported by Dickens et al. [15] we found that BTC:Lig was significantly correlated with black carbon determined by both the UV-NMR (r = 0.912, p = 0.012, n = 5) and BPCA (r = 0.883, p = 0.022. n = 5) methods. Furthermore, the OC normalized BTC was significantly correlated with BPCA (r = 0.933, p = 0.007, n = 5), but not significantly correlated with the NMR method (r = 0.737, p = 0.118, n = 5). We also found that the the BTC2:BTC of all soils were above the threshold used to distinguish burned materials from other potentially interfering compounds. Suggesting that the BTC:Lig ratio could be used to quantify the relative amounts of Py-OC in these soils. This supports the assertion that BCA COPs and BCAs recovered by the BPCA have similar sources and all of these methods (COP BCA, BPCA, and NMR) can be used to quantify relative amounts black carbon in some soils and sediment [46, 47].

### Lability of Py-OC and Lignin

We examined the lignin and Py-OC signatures before and after a 6-month incubation to examine the relative stability of these biomarkers. Interestingly, the %RY of the BDCs and BTCs decreased relative to lignin phenols as a result of the incubation. Since vascular plants are the source of lignin phenols and these were closed incubations this result was a result of either 1) the preservation of lignin relative to BDC and BTC or 2) higher rates of mineralization or transformation among these BCA classes relative to lignin. Since we did not collect reliable mass loss data for the incubations it is difficult to determine which of these explanations may be responsible for the observed trends. Either way, the lignin that remained after burning was more resistant to degradation than BDC and BTC, which is counter to widely held understanding of the relative reactivity of lignin and black carbon [30]. There may be a couple of reasons for this result. One is that residual lignin may be concentrated at the center of char particles and thereby physically protected from microbial degradation. Secondly, as previously mentioned, the CuO oxidation procedure may not be able to release BDCs and BTCs from the more recalcitrant chars (e.g. soot and turbostratic char). Therefore, the BDCs and BTCs in this study may have been derived from the more weakly bound amorphous chars or freely available [48] BCAs in the environment that may be more biologically available to mineralization or transformation. However, the major finding of our incubation was that the composition of BTCs and the relative contribution of BTC to lignin were robust indicators of Py-OC in samples that had been recently burned and experienced moderate diagenesis.

### Limitations of CuO Oxidation for the Assessment of Py-OC

Like the benzene polycarboxylic acid (BPCA) method, CuO oxidation also releases BTCs as products of oxidative hydrolysis of charred organic matter [46, 47, 49]. While the proportion BPCAs that are made up by the BTC class has been shown to decrease with burn temperature the total amount produced increased up to a burn temperature of about 600°C [49]. With increasing temperature there is an increase in the crystalline structure which probably translates to differences in the relative reactivity of the char [50] and the ability of char-derived products to be recovered by the CuO oxidation procedure. The BTCs recovered by the CuO oxidation procedure may originate as BTCs bound in those amorphous regions of char or from freely available BTCs. BTC yields from charred pine wood have been shown to peak at 300°C but decreased when temperature achieved 350°C [15]. Since the temperatures in our study did not exceed 257°C the char was likely dominated by amorphous forms of black carbon. Further research is necessary to fully determine the portion of the char macromolecule being oxidatively hydrolyized by the CuO oxidation procedure and whether this procedure is suitable for tracing higher order, more crystalline, forms of chars such as the turbostratic chars or soot. However, amorphous chars appear to dominate the black carbon continuum up to about 500°C. Which is well over the maximum temperature experienced in this study and quite a bit higher than soil temperatures measured in many wild or prescribed fires, the main exceptions being shrub land or chaparral fires and fires burning in concentrated accumulations of fuel such as logs or slash piles [32]. Therefore, BTCs recovered using CuO oxidation appear to be able trace Py-OC generated under most natural and managed fires.

## Summary and Implications

Our exploration of the response of individual and classes of CuO oxidation derived lignin and BCAs has found that lignin is consumed by burning while BCAs were preferentially preserved or produced by burning. We found that BDCs and BTCs were much more reactive to microbial degradation after burning than their lignin counterparts. However, these trends in BCAs imparted by burning survived a 6-month incubation suggesting that fire severity had first order control on BCA and lignin composition. These results also suggested that some of these compounds are robust indicators of Py-OC (in particular BTCs). We tested these ratios against samples that were specifically designed to produce a Py-OC signal without burning or charring (e.g. tannic acid) or reduce the Py-OC signal (e.g. incubated samples from this study). When combined with other CuO oxidation products, such as lignin, we found that key indicators could be used to estimate the relative contribution of Py-OC in soils.

This method may be another tool that biogeochemists could use to examine relative contributions of Py-OC to soils and sediments. Furthermore, due to the large amount of information that can be gathered from the CuO oxidation procedure this may be a useful tool for paleo-ecologists can use to assess fire frequency and fire severity regimes of past ecosystems and climate-vegetation-wildfire interactions and their subsequent interaction with carbon and sediment erosion from watersheds. Robust determinations of past fire regimes and their interactions with biogeochemical cycles may be developed when these markers and other CuO oxidation products are combined with other measures, such as charcoal and pollen records.

## Supporting Information

S1 Table Burning conditions and soil normalized values of organic matter, carbon, nitrogen, and individual biomarkers for incubated and non-incubated O and A horizon soils. Treatment = Control (C), Low (L), Moderate (M), High (H); Vanillin (Vl), Acetovanillone (Vn), Vanillic Acid (Vd), Syringealdehyde (Sl), Acetosyringone (Sn), Syringic Acid (Sd), p-Coumaric Acid (pCd), Ferulic Acid (Fd), Benzoic Acid (Bd), m-Hydroxybenzoic Acid (mBd), 3,5-Dihydroxybenzoic Acid (3,5-Bd), o-Hydroxybenzoic Acid (oBd), o-Benzenedicarboxylic acid (oBDC), m-Benzenedicarboxylic acid (mBDC), p-Benzenedicarboxylic acid (pBDC), 1,2,3-Benzenetricarboxylic acid (BTC1), 1,2,4-Benzenetricarboxylic acid (BTC2), 1,3,5-Benzenetricarboxylic acid (BTC3).
(CSV)
